# Treatment of lithium intoxication: facing the need for evidence

**DOI:** 10.1186/s40345-015-0040-2

**Published:** 2015-10-22

**Authors:** R. Haussmann, M. Bauer, S. von Bonin, P. Grof, U. Lewitzka

**Affiliations:** Department of Psychiatry and Psychotherapy, University Hospital Carl Gustav Carus, Technische Universität Dresden, Fetscherstr. 74, 01307 Dresden, Germany; Department of Internal Medicine, University Hospital Carl Gustav Carus, Technische Universität Dresden, Dresden, Germany; Mood Disorders Center of Ottawa, Ottawa, Canada

**Keywords:** Lithium, Intoxication, Affective disorders, Bipolar disorder, Treatment, Extracorporeal methods, Hemodialysis

## Abstract

Lithium has been used as the gold standard in the treatment of major depressive and bipolar disorders for decades. Due to its narrow therapeutic index, lithium toxicity is a common clinical problem. Although risk factors for lithium intoxication seem to be well-described, lacking patient education and inexperience of treatment are assumed to contribute to the probability of lithium intoxication. A review of literature shows that the treatment of lithium intoxication has not been adequately studied or standardized. The aim of this literature review is to compile and present current evidence on the treatment of lithium intoxication and contribute to a standardization regarding general treatment recommendations as well as evidence on indication for extracorporeal methods. Against the background of this common and potentially life-threatening condition, the standardization of the treatment of lithium intoxication is definitely a task for the future.

## Background

Since 1970, lithium has been approved and widely used as the gold standard for the treatment of acute episodes and maintenance treatment of bipolar disorder (Nolen 2015; Severus et al. 2014) and frequently also used in the treatment of recurrent major depressive disorders since the 1950s (Bschor 2014). In addition, lithium has been shown efficacious in augmenting response in antidepressant non-responders and in severe treatment-resistant unipolar major depression (Haussmann and Bauer 2013).

Due to a relatively narrow therapeutic index, lithium intoxication has been a common clinical problem (Timmer and Sands 1999; Hampton 2014). The effective dose range of lithium is 0.6–1.0 mmol/l, while in prolonged administration it may be toxic at 1.2 mmol/l or greater (Young 2009). Since lithium is one of the lightest elements of the periodic table, it is easily distributed throughout total body water (Young 2009; Perrone 2015). Lithium is an alkali metal, like potassium and sodium, which facilitates its rapid distribution (Young 2009). Lithium pharmacokinetics can be divided into absorption, distribution and elimination phases (Jaeger et al. 1993).

**Fig. 1:**
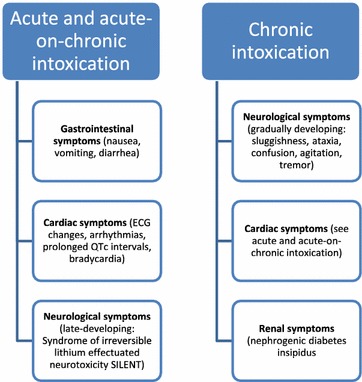
Clinical symptoms of different lithium intoxication patterns

Regarding the subtypes of lithium intoxication, there are acute, acute-on-chronic and chronic forms, which differ in their symptomatology due to lithium pharmacokinetics. Acute lithium intoxication is most often associated with gastrointestinal symptoms, cardiotoxic effects and late developing neurological signs whereas chronic forms manifest primarily as neurological symptoms, including confusion, myoclonus and seizures (Timmer and Sands 1999; Ward et al. 1994; Haussmann 2015) (Fig. 1). The rationale for clinical differences is compartment saturation. In the cases of acute lithium toxicity, lithium concentrations tend to fall rapidly due to distribution in several tissues, meanwhile chronic toxicity faces lithium-saturated tissues. For this reason, lithium toxicity depends on the exposure pattern which needs to be considered regarding treatment strategy (Waring et al. 2007).

### Risk factors for lithium intoxication

Drugs that alter renal function must be considered risk factors for lithium intoxication. In particular, angiotensin-converting enzyme inhibitors, nonsteroidal anti-inflammatory drugs and thiazide diuretics can enhance lithium serum levels by increasing renal reabsorption in the proximal tubule. General medical conditions characterized by decreased circulating volume, including viral infections with fever, gastroenteritis with diarrhea and vomiting, great heat and sauna and decreased oral intake of water augment renal reabsorption of sodium and lithium, potentially leading to toxic lithium serum levels. In this respect, nephrogenic diabetes insipidus as a common side effect of chronic lithium treatment potentially causing intoxication has to be emphasized (Timmer and Sands 1999; Erden et al. 2013). As a further potential cause of lithium intoxication suicide attempt has to be considered. Since lithium is almost exclusively excreted by the kidneys, patients with preexisting renal insufficiency are at high risk of developing lithium intoxication (Timmer and Sands 1999). Beyond that, chronic lithium treatment tends to further restrict renal function, possibly leading to a relevant limitation of lithium excretion (Boton et al. 1987; Shine et al. 2015). Nephrotoxicity of chronic lithium therapy and its management have been discussed intensely (Shine et al. 2015; Schou 1989; Severus and Bauer 2013) (Table 1).

**Table 1:** Risk factors for lithium intoxication

Drugs altering renal function (NSAID, ACE inhibitors, thiazides)
Decreased circulating volume (great heat, sauna)
Infections (viral infections, gastroenteritis with diarrhea and vomiting)
Fever
Decreased oral intake of water
Renal insufficiency
Nephrogenic diabetes insipidus
Suicide attempt

The purpose of this review is to compile and present current evidence on the treatment of lithium intoxication and contribute to a standardization of the treatment of this potentially life-threatening condition.

## Methods

A literature review, conducted using the online search engine PUBMED, targeted articles on the management of lithium intoxication. Search terms included “lithium intoxication”, “lithium intoxication management”, “lithium intoxication AND randomized, placebo-controlled trial” and “lithium intoxication AND meta-analysis”. In total, 172 single-case reports and 15 case series (with 2–10 patients each) were returned from 1955 to 11/04/2015 using the search term “lithium intoxication AND case report”, but there were no meta-analyses or randomized, placebo-controlled trials that evaluated treatment methods in the management of lithium intoxication. Thus, we focused on relevant review articles and the few available larger case series to summarize the information available about treatment decisions. The lack of background data precluded a more systematic literature review.

## Results

### Treatment of lithium intoxication: general recommendations

Lithium intoxication poses a substantial risk of permanent sequelae (Nguyen 2008). Against this background the necessity of an immediate and appropriate treatment becomes obvious. Since clinical trials dealing with treatment of lithium intoxication are lacking, current treatment guidelines of lithium intoxication are predicated on animal studies, pharmacokinetic studies and small observational studies (Wiltling et al. 2009). For this reason, the available guidelines contain an unacceptable variability of recommendations, when compared with medical treatment standards for other disturbances (Wiltling et al. 2009). As there is no specific antidote for lithium detoxification, the most effective treatment relies on minimizing exposure time to toxic lithium levels (Astruc et al. 1999).

**Fig. 2:**
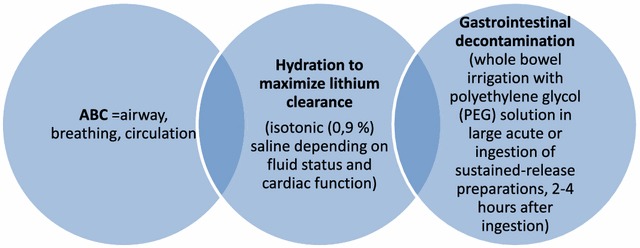
General treatment recommendations for lithium intoxication

The general approach to a lithium-intoxicated patient is similar to other poisonings, including airway management especially in cases of altered mental status, placing of a nasogastric tube and performing gastric lavage especially when patients present shortly after intoxication (Timmer and Sands 1999). Oral activated charcoal has no effect as it cannot bind lithium ions (Okusa and Crystal 1994); however, it can be important when dealing with potential intoxication from multiple substances. Lithium preparation studies recommend whole bowel irrigation using polyethylene glycol in cases of ingestion with sustained-release drugs (Okusa and Crystal 1994). Considering dehydration, volume depletion regardless of the underlying origin is a common cause of chronic lithium intoxication. A potentially existing, lithium-induced diabetes insipidus and further volume loss by gastrointestinal decontamination measures need to be stressed (Timmer and Sands 1999) and intravenous hydration should be provided with isotonic saline. In case of nephrogenic diabetes insipidus, sodium levels need to be closely monitored during intravenous hydration to prevent hypernatremia and potentially deteriorating neurological symptoms (Okusa and Crystal 1994). Addition of free water can help to prevent development of hypernatremia under such circumstances. Despite convention, forced diuresis is not able to enhance lithium excretion and is not recommended except for truly volume-depleted patients (Fig. 2) (Timmer and Sands 1999; Okusa and Crystal 1994).

### Indication for extracorporeal methods

**Fig. 3:**
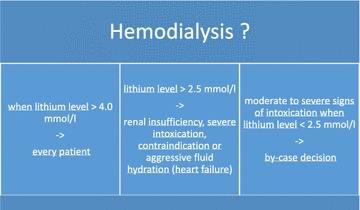
Indication for hemodialysis in lithium intoxication

Over the years lithium has proved to be one of the most readily dialyzable toxins (Okusa and Crystal 1994). Its low molecular weight (74 Da), water solubility, small volume of distribution, and insignificant protein binding determine that hemodialysis can achieve far superior lithium clearance rates compared to other detoxification methods (Bayliss 2010). But, to date, there are no consistent recommendations regarding the initiation of hemodialysis in lithium-intoxicated patient. Considering current evidence, hemodialysis should be conducted in every patient with lithium serum levels greater than 4.0 mmol/l regardless of clinical symptomatology and etiology of intoxication (Perrone 2015). When lithium levels exceed concentrations of 2.5 mmol/l, hemodialysis should be initiated when the patient suffers from severe signs of lithium intoxication, when renal impairment is on hand, when the patient underlies other conditions of limited lithium excretion and when there are other illnesses potentially deteriorating by extensive intravenous hydration (Perrone 2015). For patients not fitting one of these two categories, a decision on a by-case basis may be necessary. In such cases, a toxicologist should be consulted (Perrone 2015) (Fig. 3). Other data suggest that the decision on hemodialysis should take the type of poisoning into account since lithium kinetics seems to be of relevance regarding lithium toxicity (Jaeger et al. 1993).

The decision on hemodialysis should be determined within 8–12 h after admission (Timmer and Sands 1999; Jaeger et al. 1993). In summary, there is limited and inconsistent evidence concerning the initiation of hemodialysis in lithium-intoxicated patients. Beyond this, it seems to be debated when hemodialysis should be stopped (Perrone 2015; Lopez et al. 2012; Takahashi et al. 2011; Meertens et al. 2009). In particular, the rebound phenomenon is the major concern (Timmer and Sands 1999; Perrone 2015; Okusa and Crystal 1994; Bayliss 2010). After initiation of hemodialysis, blood lithium levels tend to decline rapidly, but can increase as re-equilibration from the extracellular site takes place (Bayliss 2010). For that reason serial measurement of lithium levels is inevitable (Timmer and Sands 1999; Okusa and Crystal 1994). From the clinical point of view, the values provided by the serial measurement are most informative. The sequence of values helps differentiating whether the high level is due mainly to an absorption peak and quickly drops off, or whether it persists or even rebounds, demonstrating a marked, chronic saturation of lithium in the tissues.

Lithium concentrations should be measured every 2–4 h initially to evaluate treatment efficacy until concentrations approach therapeutic levels (Perrone 2015). If this is the case or if lithium serum levels constantly trend downward, patient lithium concentrations can be measured less frequently (i.e., every 6–12) until symptoms of intoxication resolve (Perrone 2015). The rebound phenomenon necessitates repeated dialysis sessions in cases of severe lithium intoxication (Timmer and Sands 1999; Okusa and Crystal 1994), while high lithium levels at admission coupled with low initial creatinine clearance and low blood sodium concentration at admission seem to be associated with a greater number of required dialysis sessions (Lopez et al. 2012). To control the rebound phenomenon, dialysis should be repeated if necessary until lithium levels remain below 1.0 mmol/l for 6–8 h after treatment (Perrone 2015). A recent publication describes sequential procedures of hemodialysis for reducing lithium levels below 1.0 mmol/l followed by continuous veno-venous hemofiltration for preventing rebound of lithium concentration as a further effective method to encounter danger of rebounding lithium serum levels (Meertens et al. 2009). Since extracorporeal methods typically result in circulatory stress, alternative methods to hemodialysis might be especially useful in hemodynamically unstable patients (Perrone 2015).

## Discussion

From the clinical perspective, the prevention of lithium intoxication should focus both on patient and physician-related factors. A review of case series of intoxications treated in a hemodialysis unit showed that lithium intoxication is largely a therapeutic oversight—it is mostly preventable because the vast majority of cases were due to treatment based on inexperience or because patients lacked sufficient education about the principles of lithium maintenance. In these cases, hemodialysis was indicated when both the clinical symptoms of intoxication were evident in the patient and serial lithium levels were dropping discernibly more slowly than one would expect from lithium’s pharmacokinetics. Unfortunately, in many medical schools the education of physicians about lithium treatment is no longer included in the curriculum and consequently many psychiatric residents complete their education without treating a single patient with lithium. Given the value and potential lithium holds this is most unwise.

The main patient-related factor that places a patient at an increased risk for lithium intoxication, thus, might be summarized as a lack of lithium-related knowledge, which is known to be negatively correlated with age, whereas duration of treatment, sex, education and diagnosis does not seem to be related to lithium-specific knowledge (Schaub et al. 2001). Surprisingly, lithium-related knowledge has been shown to be insufficient even in a highly selected and compliant outpatient clinic cohort (Schaub et al. 2001). Consequently, intensifying patient education, especially in older patients taking lithium, on a regular basis seems to be most promising in potentially preventing lithium intoxications regarding patient-related factors. Despite the patients’ education, other strategies of preventing lithium intoxication like dosing considerations should be included (Malhi and Tanious 2011). In addition, a positive influence on the poor compliance in long-term lithium use might be a positive side effect in a less frequent administration (Malhi and Tanious 2011).

Recommendations for the safe use of lithium include patient’s selection/indication, careful screening procedure, examinations before and during lithium therapy and clinical and laboratory monitoring (including side effects). Lithium dosage needs to be individually tailored. More importantly are all psychological interventions known to be effective in preventions of non-compliance (Table 2) (Vestergaard et al. 2006).

**Table 2:** Psychological interventions for prevention of non-compliance

Psychoeducation about affective disorders including patients concepts and misconcepts (e.g. regarding pharmacotherapy)
Education about potential risks associated with lithium withdrawal
Importance of other influences such as illegal drugs and alcohol
Detection of warning signs or early symptoms
Education of relatives and caregivers (including general practitioner)

In summary there is insufficient information and inconsistent data concerning the indications and termination of hemodialysis in lithium-intoxicated patients. New sequential procedures with less stressful circulatory effects based on theoretical consideration of lithium pharmacokinetics are proposed, but need to be validated in larger studies. With regard to lithium intoxication, additional clinical observations, more comprehensive analyses and prospective treatment studies are urgently needed. To date, we only have available case reports, limited case series, expert opinions and some small retrospective analyses in cases which, against the background of a frequent and potentially life-threatening condition, are insufficient. Future research should aim at the development of standards for a differentiated lithium intoxication treatment.
